# Stimulation of Synaptic Vesicle Exocytosis by the Mental Disease Gene *DISC1* is Mediated by N-Type Voltage-Gated Calcium Channels

**DOI:** 10.3389/fnsyn.2016.00015

**Published:** 2016-06-14

**Authors:** Willcyn Tang, Jervis Vermal Thevathasan, Qingshu Lin, Kim Buay Lim, Keisuke Kuroda, Kozo Kaibuchi, Marcel Bilger, Tuck Wah Soong, Marc Fivaz

**Affiliations:** ^1^DUKE-NUS Medical School, Program in Neuroscience and Behavioral DisordersSingapore, Singapore; ^2^Department of Physiology, Yong Loo Lin School of Medicine, National University of SingaporeSingapore, Singapore; ^3^Department of Cell Pharmacology, Nagoya University Graduate School of MedicineNagoya, Japan; ^4^DUKE-NUS Medical School, Program in Health Services and Systems ResearchSingapore, Singapore

**Keywords:** DISC1, hippocampus, glutamate, psychiatric disorders, schizophrenia, neurotransmitter, synaptic vesicle release

## Abstract

Lesions and mutations of the DISC1 (Disrupted-in-schizophrenia-1) gene have been linked to major depression, schizophrenia, bipolar disorder and autism, but the influence of DISC1 on synaptic transmission remains poorly understood. Using two independent genetic approaches—RNAi and a DISC1 KO mouse—we examined the impact of DISC1 on the synaptic vesicle (SV) cycle by population imaging of the synaptic tracer vGpH in hippocampal neurons. DISC1 loss-of-function resulted in a marked decrease in SV exocytic rates during neuronal stimulation and was associated with reduced Ca^2+^ transients at nerve terminals. Impaired SV release was efficiently rescued by elevation of extracellular Ca^2+^, hinting at a link between DISC1 and voltage-gated Ca^2+^ channels. Accordingly, blockade of N-type Cav2.2 channels mimics and occludes the effect of DISC1 inactivation on SV exocytosis, and overexpression of DISC1 in a heterologous system increases Cav2.2 currents. Collectively, these results show that DISC1-dependent enhancement of SV exocytosis is mediated by Cav2.2 and point to aberrant glutamate release as a probable endophenotype of major psychiatric disorders.

## Introduction

Genome-wide association studies and exome sequencing efforts have led to the identification of hundreds of variants associated with psychiatric disorders (International Schizophrenia et al., 2009; Moskvina et al., 2009; Glessner et al., 2010; Fromer et al., 2014; Schizophrenia Working Group of the Psychiatric Genomics, 2014), confirming the complex polygenic nature of these diseases. These genomic data also revealed a significant overlap in genes or gene networks associated with distinct mental illnesses (Cross-Disorder Group of the Psychiatric Genomics et al., 2013), suggesting a common genetic and perhaps circuitry basis for major psychiatric disorders. However, the synaptic and circuitry defects underlying these disorders remain poorly defined, hindering the development of therapeutic solutions.

DISC1 is the prototypical example of a gene associated with several major psychiatric disorders. It was discovered in a Scottish family at the site of a balanced chromosomal translocation that strongly segregates with major depression, schizophrenia and bipolar disorder (St Clair et al., 1990; Millar et al., 2000, 2001). The high penetrance (~70%) of this translocation for mental illness supports a causal link between this genetic lesion and major psychiatric conditions (Chubb et al., 2008). DISC1 variants (haplotypes, single nucleotide polymorphisms and copy number variations) have since been independently associated with depression, schizophrenia, bipolar disorders and autism spectrum disorders (Ekelund et al., 2001, 2004; Sachs et al., 2005; Kilpinen et al., 2008). Thus, DISC1 is a major susceptibility factor for mental illness and a relevant genetic entry point to identify core endophenotypes implicated in neuropsychiatric disorders.

The translocation breakpoint in this Scottish family is located in the C-terminal portion of DISC1 and results in overall reduced expression of the full-length transcript and protein (Millar et al., 2005), suggesting that haploinsufficiency is the main mechanism by which this chromosomal alteration confers risk to disease. Alternatively, it has been proposed that a C-terminal truncated form of DISC1 is expressed from the translocated allele and may be pathogenic (Hikida et al., 2007; Pletnikov et al., 2008), although expression of the truncated DISC1 protein in translocation carriers remains to be demonstrated. Consistent with a disease mechanism based on DISC1 loss-of-function, DISC1 expression is also attenuated in human induced pluripotent stem (iPS) cells derived from members of a family with a DISC1 frame-shift mutation that co-segregates with major psychiatric disorders (Wen et al., 2014).

The identification of a large DISC1 interactome consisting of proteins belonging to different ontologic families suggests broad functions of DISC1 in nerve cells. Accordingly, DISC1 has been implicated in multiple aspects of neuronal and brain development, including neurogenesis (Clapcote et al., 2007; Shen et al., 2008; Mao et al., 2009; Singh et al., 2010; Lee et al., 2011), neuronal migration (Kamiya et al., 2005; Duan et al., 2007; Kubo et al., 2010; Steinecke et al., 2012) and maturation (Duan et al., 2007; Shinoda et al., 2007; Niwa et al., 2010). Even though DISC1 interacts with several signaling proteins known to regulate synaptic functions, relatively little is known about functions of DISC1 at the synapse. In particular, the impact of DISC1 on neurotransmitter release remains largely unexplored, despite the fact that aberrant dopamine and glutamate neurotransmission is a probable cause of schizophrenia and other mood disorders (Howes et al., 2015).

Given the preferential expression of DISC1 in the hippocampus and the involvement of this brain structure in cognition and psychiatric disorders (Chubb et al., 2008), we set out to determine the impact of DISC1 on synaptic vesicle (SV) cycling in hippocampal neurons. We used two independent genetic strategies to alter DISC1 expression—RNAi and a DISC1 KO mouse—and imaged SV cycling and Ca^2+^ dynamics in large synapse populations. We show that DISC1 elevates synaptic Ca^2+^ signals and boosts SV exocytosis at glutamatergic terminals. Our results further indicate that N-type voltage-gated Ca^2+^ channels (VGCCs) mediate the stimulatory effect of DISC1 on SV release. These findings identify a central role of DISC1 in neurotransmitter release and provide new insights on the biological basis of synaptic dysfunction in major psychiatric disorders.

## Materials and Methods

### DNA, shRNA Constructs, Lentiviruses and Antibodies

The pCAGGs vGlut1-pHluorin (vGpH) and pCAGGs Synaptophysin-GCaMP3 (SyGC3) constructs were gifts from R. Edwards (UCSF; Voglmaier et al., 2006) and S. Voglmaier (UCSF; Li et al., 2011). The pFUGW (Addgene #14883) shRNA-expressing lentiviral vector was modified to express mCherry (pFUmChW). The shRNA targeting sequences are as follows: (1) Scramble 5′GGAGCAGACGCTGAATTAC3′ (Kamiya et al., 2005); (2) DISC1-E 5′GGCTACATGAGAAGCACAG3′ (exon 2; Duan et al., 2007); and (3) DISC1-A 5′GGAAGGGCTAGAGATGTTC3′ (exon 9) designed with Block-it shRNA from Invitrogen. pFUGW scramble shRNA was a gift from A. Sawa (Johns Hopkins). The DISC1 shRNAs constructs were cloned by introducing double-stranded DNA oligos into lentiviral vector pll3.7 (Addgene #11795) using the HpaI and XhoI sites. DNA fragments containing the U6 promoter and shRNAs were then PCR amplified and cloned into pFUmChW using the PacI site. The human DISC1 gene L variant (NCBI Refseq NM018662.2) was PCR amplified from pCMV6-XL5 DISC1-tGFP (Origene) and cloned into pIRES2-DsRed-Express (Clontech) using NheI and SmaI sites. All constructs were sequenced before use. Lentiviral particles expressing pFUmChW shRNAs were prepared as described in Tiscornia et al. (2006). The DNA constructs used for whole-cell patch clamp recording are as follows: Cav2.1 (generated in T. W. Soong’s lab), Cav2.2 (Addgene #26568), GFP-β_2a_ and α_2_δ_1_ (kindly provided by T. Snutch, UBC). The rabbit polyclonal Abs against Cav2.1 (#ACC-001) and Cav2.2 (#ACC-002) were from Alomone Labs. The polyclonal Ab against the C-terminus of mouse DISC1 was previously described (Kuroda et al., 2011). The polyclonal Ab against human DISC1 (ab59017) and monoclonal Ab against bassoon were from Abcam (ab82958).

### Mouse Lines, Primary Neuron Cultures and Transfection Protocols

*DISC1* (Δ2–3) mice (C57BL/6JJmsSlc) have been described before Kuroda et al. (2011). Heterozygous *DISC1*^wt/Δ2–3^ mice were crossed to each other to obtain *DISC1*^wt/wt^ and *DISC1*^Δ2–3/Δ2–3^ lines from which hippocampal neurons were prepared from E17/E18 embryos, according to published procedures using papain (Worthington) digestion (Huettner and Baughman, 1986). Primary rat hippocampal neurons were prepared from E18 embryos according to Kaech and Banker (2006) and as previously described in Garcia-Alvarez et al. (2015). For live-cell imaging and immunocytochemistry experiments, neurons were grown on poly-L-Lysine coated glass coverslips, on top of a glial feeder according to the Banker Protocol (Kaech and Banker, 2006). For biochemical analysis, cells were seeded on poly-L-Lysine coated 6-well culture dishes. Unless otherwise stated, neurons were cultured for 14–16 days. The vGpH and SyGC3 constructs were electroporated in freshly-dissociated neurons using the Nucleofector kit (Amaxa Biosystems, Lonza). Lentiviral particles expressing shRNAs were added on DIV2, at a MOI (multiplicity of infection) of 1–3. All animal procedures were approved by the SingHealth Institutional Animal Care and Use Committee (IACUC) of Singapore.

### Live-Cell Confocal Imaging and Immunofluorescence

Time-lapse confocal imaging was performed on an inverted Eclipse TE2000-E microscope (Nikon, USA), mounted with a spinning-disk confocal scan head (CSU-10; Yokogawa, Japan), and equipped with a temperature controlled (36.5°C) stage and an autofocusing system (PFS; Nikon). Images were acquired with an Orca-Flash 4.0 CCD camera (Hamamatsu Photonics, Japan) controlled by MetaMorph 7.8.6 (Molecular Devices, CA, USA) at 0.5 Hz or 20 Hz. Samples were imaged using a 60× (NA 1.4) objective in Tyrode’s buffer (150 mM NaCl, 2.5 mM KCl, 1 mM CaCl_2_, 1 mM MgCl_2_, 6 mM glucose, 25 mM HEPES, pH 7.4) supplemented with 25 μM 6-cyano-7-nitroquinoxaline-2,3-dione/CNQX (Tocris Bioscience) and 50 μM D,L-2-amino-5-phosphonovaleric acid/AP5 (Tocris Bioscience). Coverslips were mounted in an RC-21BRFS chamber (Warner Instruments, USA) equipped with platinum wire electrodes. Field stimulation was induced by a square pulse stimulator (Grass Technologies, USA) and monitored by an oscilloscope (TDS210, Tektronix, USA). Trains of action potentials (APs) were generated by applying 20 V pulses (1 ms duration) at 10 or 20 Hz. Our typical stimulation paradigm for vGpH measurements involved two consecutive trains of 300 APs at 10 Hz, separated by ~5 min to allow synapses to recover. vGpH responses between the first and second stimulation were highly reproducible (data not shown). For measuring SV exocytic rates, the second stimulation was preceded (30 s earlier) by the addition of Bafilomycin A1 (Baf; AG scientific, USA) 0.5 μM. To normalize for total expression of vGpH in each individual bouton, 50 mM NH_4_Cl was added at the end of the time series. For SyGC3 measurements, Ca^2+^ signals were normalized by adding 10 μM or 50 μM (for Figure 4G) ionomycin (Sigma-Aldrich) at the end of the stimulation protocol. ω-agatoxin TK125 nM (Tocris Bioscience) and ω-conotoxin GVIA 125 nM (Alomone Labs) were used for experiment involving inhibition of P/Q-type Ca^2+^ channels and N-type Ca^2+^ channels activity, respectively. For immunofluorescence studies, neurons grown on glass coverslips were fixed in 4% paraformaldehyde with 4% sucrose in PBS and permeabilized with 100 ng/ml Digitonin (Sigma-Aldrich). Cells were then incubated with 5% goat serum to block non-specific binding sites and stained with the primary and Alexa Fluor-conjugated secondary antibodies (Life Technologies). Coverslips were then mounted on glass slides and imaged with an inverted laser scanning confocal microscope (LSM710, Zeiss) with a Plan-Apochromat 63× (NA = 1.40) objective.

### Image Analysis

**Figure 1:**
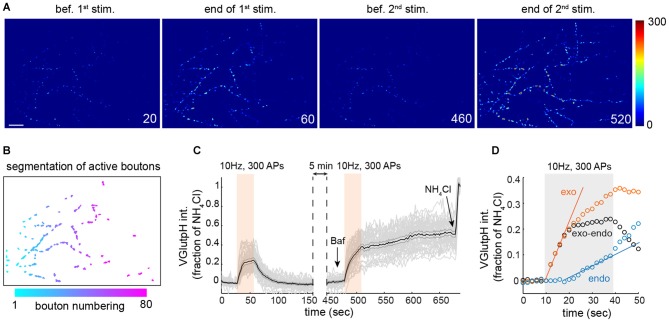
**Extracting synaptic vesicle (SV) exo- and endocytic rates by population imaging of vGpH. (A)** Snapshots of rat hippocampal neurons (DIV 15) expressing vGpH and stimulated with two consecutive trains of action potentials (APs; 300 APs, 10 Hz) before (first two images) and after (last two images) addition of Bafilomycin (Baf). Time is in sec. scale bar: 10 μm. **(B)** Automated segmentation of active boutons based on vGpH responses after the first stimulation. **(C)** Individual (gray) and average vGpH (black) traces derived from segmented boutons. Shaded errorbar indicates 95% confidence interval. **(D)** Measurement of exo- and endocytic rates based on vGpH responses before and after Baf (see text).

For automated analysis of vGpH responses, we wrote a Matlab script that segments responsive boutons based on the difference between peak vGpH intensity during the first stimulation and baseline intensity prior to stimulation (Δ*F* = *F*_peak_ − *F*_baseline_). An intensity threshold for Δ*F* was selected to resolve individual boutons and exclude those with Δ*F* below 5%. The same threshold was used for all conditions in one independent experiment (i.e., one neuron preparation with control vs. DISC1-depleted conditions). This threshold value was minimally adjusted (less than 10% change) across all independent experiments described in this article. Binarized boutons were then slightly dilated (Figure 1B) to ensure that vGpH fluorescence was captured in its totality even after minor lateral movement or change in shape. Time series with minor *x–y* drifts (originating from the stage) were re-aligned using a script previously described (Thevathasan et al., 2013). Segmented boutons were then size gated, with gating parameters kept constant across all experiments. vGpH fluorescence intensity was then extracted in each segmented bouton across the time series and divided by the signal after NH_4_Cl to normalize for vGpH expression (Figure 1C). This segmentation strategy ensures that the same boutons are analyzed during the two consecutive AP trains. vGpH traces with significant baseline drifts between the first and second stimulation or after Baf application were excluded. A similar segmentation approach was used to analyze SyGC3 Ca^2+^ signals. SV exocytic rates were obtained by measuring the slope of the vGpH rise during the second stimulation (in the presence of Baf). The first six time points (during stimulation) were used for linear regression analysis. To measure endocytic rates, the vGpH trace during the first stimulation was subtracted from that obtained during the second stimulation. The resulting trace is a measure of endocytosis (Figure 1D). Endocytic rates were measured by linear fitting of six time points chosen after stimulation onset when endocytosis kicks in. Presynaptic localization, abundance and density of Cav2.1 or Cav2.2 were analyzed with a modified version of a Matlab script described previously (Poon et al., 2014). All Matlab scripts are available upon request.

### Immunoblotting

Cultured primary neurons or HEK293T cells were washed with ice-cold PBS and lysed with RIPA buffer (10 mM Tris-HCl pH = 7.2, 150 mM NaCl, 1% TritonX-100, 0.1% SDS, 5 mM EDTA, 0.25% Na-deoxycholate) supplemented with Complete protease inhibitor and PhosphoStop phosphatase inhibitor (Roche). For analysis of hippocampal tissue, the hippocampi from P10 mice were harvested and homogenized in RIPA buffer using a Dounce tissue homogenizer. Lysates were cleared by centrifugation and boiled in Laemmli sample buffer. Equal amount of total proteins were loaded. Samples were then analyzed by SDS-PAGE, transferred onto nitrocellulose membranes, probed with appropriate primary and HRP-labeled secondary antibodies and revealed by enhanced chemiluminescence.

### Whole-Cell Patch Clamp Recordings of Cav2.1 and Cav2.2 Currents

Cav2.1 or Cav2.2, the auxiliary subunits (GFP-β_2a_ and α_2_Δ_1_) and DISC1 were transiently transfected in HEK 293 cells using the calcium phosphate method (Huang et al., 2012). Whole-cell patch-clamp recordings were performed within 36–72 h after transfection. The external solution contained (in mM) 10 HEPES, 140 TEA-MeSO_3_ and 5 BaCl_2_ (pH 7.4, 300–310 mOsm). The glass pipette solution was backfilled with pipette solution (in mM) 10 HEPES, 5 CsCl, 138 Cs-MeSO_3_, 0.5 EGTA, 1 MgCl_2_, 2 mg/ml MgATP (pH 7.3, 290–300 mOsm). HEK 293 cells were held at −90 mV using the Axopatch 700B amplifier (Axon Instruments). The series resistance for all recordings was less than 5 MΩ; 70–80% compensation on serial resistance and cell membrane capacitance were applied. A P/4 protocol was used to subtract leakage current. All recordings were obtained with an Axon Digidata 1440A data acquisition system, sampled at 5–50 kHz and low pass-filtered at 1 kHz or 6 kHz. The I-V curves were obtained from 10 mV voltage-steps ranging from −50 to 40 mV and fitted with a modified Boltzmann equation:

$$I\;=\;G_{\text{max}}(E_{\text{rev}}-V)/\left(1+e^{\frac{V_{1/\text{2act}}-V}{k_{\text{act}}}}\right)$$

where, *I* = current density (in pA/pF), *G*_max_ = maximum conductance (in nS/pF), *E*_rev_ = reversal potential, *V* = measured potential, *V*_1/2act_ = midpoint voltage for current activation, and *k*_act_ = the slope factor.

We used a tail protocol to measure current density; cells were depolarized using 10 mV voltage steps, from −60 to 60 mV. Following depolarization, tail currents were evoked with a 10 ms pulse at −50 mV. The data were fitted with single Boltzmann equation:

$$I\;=\;I_{\;\text{min}}+(I_{\text{max}}-I_{\;\text{min}})/\left(1+e^{\frac{V_{\text{1/2inact}}-V}{k_{\text{inact}}}}\right)$$

where, *I*_max_ and *I*_min_ = maximal and minimal current respectively, *V*_1/2inact_ = the half-maximal voltage for current inactivation, *k*_inact_ = slope of inactivation curve.

### Statistics

The experimental design of this study implies that data collected at individual synaptic terminals are clustered according to neuron preparations and imaged fields. Galbraith et al. (2010) demonstrated that such clustering can adversely affect statistical inference when not accounted for. In order to probe for clustering effects (i.e., intra-field correlations), we tested whether bouton responses significantly vary from field to field. For this, we conducted Wald tests of the null hypothesis of no difference in mean outcome across fields within each condition, which revealed (*p*-value < 0.0001) strong intra-field correlation. To account for these correlations, we performed our statistical analysis in the framework of linear mixed models (Laird and Ware, 1982) with normally-distributed random field effects and preparation fixed effects. In experiments involving one genetically-modified condition and one control group, we tested the null hypothesis of no difference between the mean outcome of the groups via 2-sample *t*-tests. In experiments involving two genetically-modified conditions and one control group, we tested the null hypothesis of no difference between the mean outcome of each group and the control group jointly using Wald tests. All tests were performed at the 5% level of statistical significance and carried out using the statistical software Stata version 13.2. Because our statistical approach (linear mixed model) is not a standard practice when analyzing synaptic properties, we compared the p-values obtained with our method with those measured by the more common field averaging approach, where the information of a field is collapsed to a single independent observation by taking the mean of bouton responses. Both methods are valid and gave comparable results (Supplementary Table T1), although the field averaging approach is less statistically efficient as it diminishes the information that can be obtained from the data by reducing individual measurements in a field to one observation (Galbraith et al., 2010).

## Results

### DISC1 Loss-of-Function Slows Down SV Cycling

We opted for an imaging approach based on the synaptic tracer vGpH to explore the impact of DISC1 on the SV cycle. vGpH consists of the pH-sensitive GFP variant pHluorin (Miesenbock et al., 1998) fused to the SV-resident glutamate transporter vGlut1. pHluorin, which faces the acidic lumen of SVs, undergoes a ~20-fold increase in fluorescence intensity when exposed to the neutral pH of the extracellular milieu after membrane fusion. (Sankaranarayanan et al., 2000). Following glutamate discharge and vGpH re-uptake, SVs are rapidly re-acidified and vGpH fluorescence is quenched. This property has made vGpH a valuable tool to monitor both exo- and endocytosis of SVs at single synapses.

Due to the inherent variability in the properties of individual synaptic boutons (Ariel et al., 2013) we measured SV cycling in large synapse populations. For this, we developed an image analysis algorithm that identifies all responding boutons in a given field (Figures 1A,B), and imaged 16–18 fields from at six independent rat hippocampal neuron cultures, yielding close to a thousand synaptic boutons for each condition. We employed this algorithm to monitor SV cycling in response to a stimulation paradigm that consists of two consecutive trains of APs (Fernandez-Alfonso and Ryan, 2004; Burrone et al., 2006). The amplitude of the first vGpH transient is governed by the relative rates of SV exo- and endocytosis during stimulation (Figure 1C). To separate the contributions of exo- and endocytosis, we blocked SV re-acidification with the vacuolar H^+^ ATPase inhibitor Bafilomycin (Baf) during the second stimulation, which allows selective measurement of SV exocytosis (Figure 1C). To account for cell-to-cell variation in vGpH expression, we normalized each trace based on the vGpH signal measured after NH_4_Cl addition (Figure 1C). The endocytic component of SV cycling was then computed by subtracting the first vGpH transient from the vGpH response after Baf treatment (Figure 1D). Synaptic traces with baseline drifts between the first and second stimulation or after Baf addition were excluded from the analysis (see “Materials and Methods” Section). Exocytosis largely dominates at the onset of stimulation, while endocytosis kicks in towards the end of the AP train. Exo- and endocytic rates were finally obtained by linear fitting of the exo- and endo traces (Figure 1D). Exocytic rates are about three times higher than endocytic rates under this stimulation protocol. To evaluate the variability of bouton responses across fields and neuron preparations, we compiled exocytic rates from six independent experiments (Supplementary Figure S1). Statistical analysis of these responses revealed substantial field to field variation (see “Materials and Methods” Section), implying that the information collected from each bouton does not constitute an independent measurement. To compare synaptic responses in different fields and conditions we used a statistical approach that accounts for correlations within fields and variations in neuron preparations (see “Materials and Methods” Section and Supplementary Table T1).

**Figure 2:**
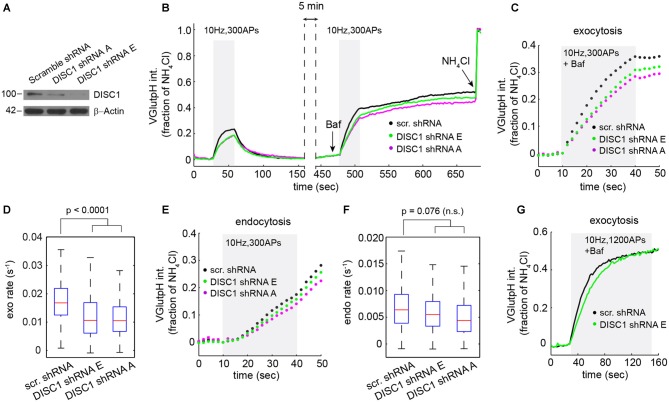
**DISC1 silencing by RNAi slows down SV exocytosis. (A)** Immunoblot analysis of DISC1 in mouse hippocampal neurons (DIV 8) transduced with scramble, DISC1-A and DISC1-E shRNAs. **(B)** Average vGpH traces derived from neurons expressing scr (1204 boutons, 18 fields), -E (960 boutons, 18 fields) and -A (924 boutons, 16 fields) shRNAs from six independent experiments. **(C,D)** Analysis of SV exocytosis after Baf treatment. **(C)** Exocytic profiles of neurons expressing the indicated shRNAs. **(D)** Boxplot of exocytic rates. **(E,F)** Analysis of SV endocytosis. **(E)** Endocytic profiles. **(F)** Boxplot of endocytic rates. **(G)** Exocytic profile of cells expressing the indicated shRNAs in response to a stimulation (1200 APs, 10 Hz) that depletes the total releasable pool.

We then silenced the DISC1 gene using a published shRNA sequence (shRNA-E), targeting exon 2 (Duan et al., 2007) and a new shRNA sequence (shRNA-A) targeting exon 9. These two shRNAs target regions of the *DISC1* gene that are fully conserved in rodents and are thus expected to silence rat and mouse *DISC1* with the same efficiency. To probe the effect of these shRNAs on DISC1 protein levels, we used an antibody raised against the C-terminus of mouse DISC1, which has previously been validated in a *DISC1* KO mouse (Kuroda et al., 2011). In our hands, this antibody poorly reacts with rat DISC1 (not shown). In mouse hippocampal neurons, however, it detects one major protein at ~100 kDa corresponding to the predicted full length DISC1 (Kuroda et al., 2011) and which is substantially reduced in both shRNA-E and -A transduced cells (Figure 2A).

Next, we probed the impact of these shRNAs on the SV cycle in rat hippocampal neurons. We initially chose to work with rat, rather than mouse neurons, because we can harvest substantially more cells from a rat embryonic litter. vGpH signals in shRNA-E and -A expressing neurons display a slower rise and lower amplitude during the first and second stimulation, relative to control cells expressing a scramble shRNA (Figure 2B). Analysis of exocytosis shows substantial synapse-to-synapse variation in all conditions, but reveals a marked decrease in exocytic rates (Figures 2C,D) and amplitudes (Figure 2C) in both shRNA-E and -A expressing neurons. DISC1 silencing also resulted in slightly slower SV endocytosis, although this effect did not reach statistical significance (Figures 2E,F).

Reduced amplitude of the exocytic response prompted us to test whether DISC1 also regulates the size of the total releasable pool. For this, we applied a stimulus strong enough (1200 APs at 10 Hz) to maximally deplete releasable SVs from presynaptic terminals (Ariel and Ryan, 2010). Under this stimulation regime, vGpH responses reached the same plateau in DISC1-silenced and control neurons, albeit with different kinetics, arguing against an effect on the total releasable pool but confirming the stimulatory function of DISC1 in SV exocytosis (Figure 2G).

**Figure 3:**
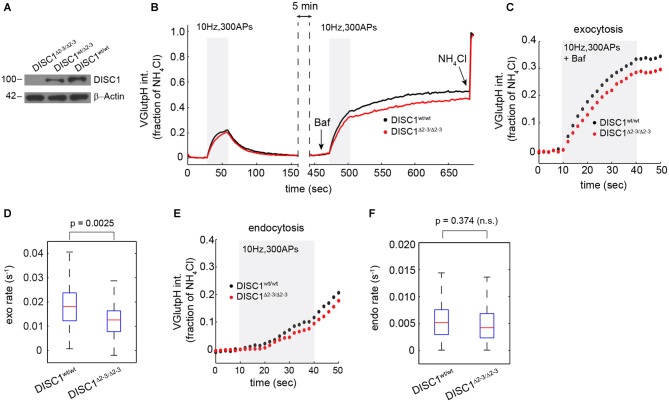
**Attenuated SV release in hippocampal neurons from *DISC1*^Δ2–3/Δ2–3^ mice. (A)** Immunoblot analysis of DISC1 in hippocampal lysates prepared from P10 *DISC1*^Δ2–3/Δ2–3^, *DISC1*^wt/Δ 2–3^ and *DISC1*^wt/wt^ mice, confirming the ablation of full-length DISC1 (~100 kD). **(B)** Average vGpH traces in *DISC1*^wt/wt^ (575 boutons, 7 fields) and *DISC1*^Δ2–3/Δ2–3^ (682 boutons, 9 fields) neurons in response to two consecutive trains of APs and obtained from two independent experiments. **(C,D)** Analysis of SV exocytosis from vGpH responses after Baf. Average kinetics **(C)** and rates **(D)** of vGpH exocytic responses. **(E,F)** Analysis of SV endocytosis. Average kinetics **(E)** and rates **(F)** of vGpH endocytic responses.

To rule out the possibility of RNAi off-target effects—shRNA-E has recently been suggested to inhibit neuron migration in the developing cortex independently of DISC1 (Tsuboi et al., 2015)—we examined SV cycling in a *DISC1* KO mouse that lacks exons 2 and 3 of the *DISC1* gene (Kuroda et al., 2011). Homozygous *DISC1*^Δ2–3/Δ2–3^ mice show no detectable levels of the major isoform of DISC1 (Figure 3A). Hippocampal neurons derived from *DISC1*^Δ2–3/Δ2–3^ mice display SV cycling defects that are remarkably similar to these observed with RNAi. The rates and amplitudes of exocytic responses were substantially reduced relative to wt cells (Figures 3B–D), while endocytic rates were marginally, but not significantly diminished (Figures 3E,F). Together, these results show that both genetic ablation and RNAi knockdown of DISC1 selectively disrupts SV exocytosis at glutamatergic synapses with no detectable impact on the total releasable pool.

### DISC1 Stimulates Ca^2+^ Influx and Regulates Cav2.2-Dependent SV Release

**Figure 4:**
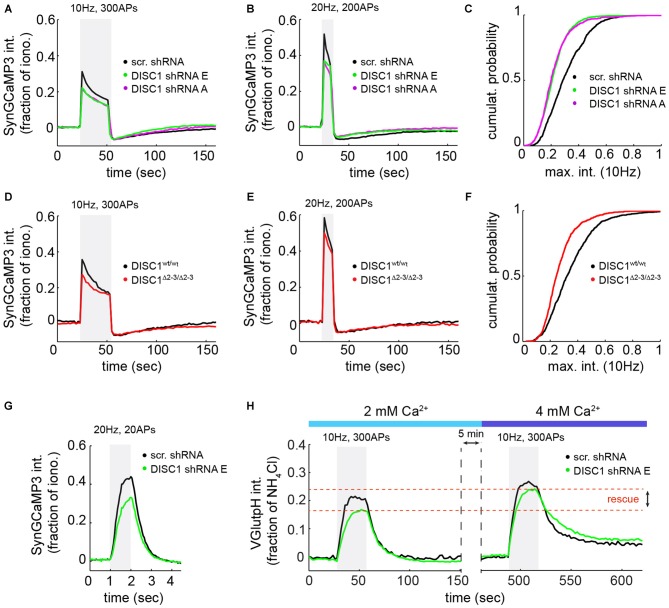
**DISC1 loss-of-function reduces evoked Ca^2+^ transients at nerve terminals**. SyGC3 imaging in rat hippocampal neurons (DIV14–16) in response two different trains of APs. **(A)** Average Ca^2+^ transients in neurons expressing scr (1052 boutons, 15 fields, 5 experiments (exps)), DISC1-E (1836 boutons, 9 fields, 3 exps) and -A (754 boutons, 11 fields, 3 exps) shRNAs, in response to 300 APs, 10 Hz. The DISC1-E and DISC-A groups are significantly different than the scr group (*p* = 0.0057). **(B)** Average Ca^2+^ transients in neurons expressing scr (861 boutons, 9 fields, 5 exps), DISC1-E (2118 boutons, 7 fields, 3 exps) and -A (1549 boutons, 9 fields, 3 exps) shRNAs, in response to 200 APs, 20 Hz. **(C)** Cumulative probability of SyGC3 peak intensity from individual boutons corresponding to **(A)**. **(D)** Average Ca^2+^ transients in *DISC1^wt/wt^* (1090 boutons, 11 fields, 3 exps) and *DISC1*^Δ2–3/Δ2–3^ (727 boutons, 9 fields, 3 exps) neurons, in response to 300 APs, 10 Hz. The *DISC1^wt/wt^* and *DISC1*^Δ2–3/Δ2–3^ groups are statistically different (*p* = 0.0182). **(E)** Average Ca^2+^ transients in *DISC1^wt/wt^* (1365 boutons, 9 fields, 3 exps) and *DISC1*^Δ2–3/Δ2–3^ (1233 boutons, 8 fields, 3 exps) neurons, in response to 200 APs, 20 Hz. **(F)** Cumulative probability of SyGC3 peak intensity from individual boutons corresponding to **(D)**. **(G)** Average Ca^2+^ transients in neurons expressing scr (290 boutons, 6 fields, 2 exps), and DISC1-E (390 boutons, 6 fields, 2 exps) shRNAs, in response to 20 APs, 20 Hz. The scr and DISC1-E groups are statistically different (*p* = 0.0036). **(H)** Average vGpH traces in scr (180 boutons, 5 fields, 2 exps) and DISC1-E (488 boutons, 6 fields, 2 exps) shRNA-expressing neurons during two consecutive trains of APs (300 AP, 10 Hz) in the presence of 2 or 4 mM extracellular Ca^2+^.

Because SV exocytosis is initiated by Ca^2+^ influx through VGCCs, we measured evoked Ca^2+^ transients at presynaptic terminals using the SV-targeted Ca^2+^ sensor SyGC3 (Li et al., 2011). Ca^2+^ signals evoked by 300 APs (10 Hz) or 200 APs (20 Hz) show a rapid rise and partial decay during the stimulus (Figures 4A,B,D,E), as observed by others (Li et al., 2011). These Ca^2+^ transients were significantly reduced in shRNA-E and -A expressing neurons, under the same stimulation conditions used for vGpH measurements (Figures 4A,C), or in response to a higher frequency stimulus (Figure 4B). Similarly, *DISC1*^Δ2–3/Δ2–3^ neurons show lowered Ca^2+^ transients compared to wt cells (Figures 4D–F). We were concerned that, under such prolonged stimulations, Ca^2+^ concentration in terminals might reach levels that partially saturate SyGC3. Ca^2+^ signals were therefore also examined in response to shorter stimulations (20 APs, 20 Hz) under conditions well below SyGC3 saturation (Tian et al., 2009; Akerboom et al., 2012). A clear decrease in the amplitude of Ca^2+^ transients was also observed in DISC1-silenced neurons under this stimulation regime (Figure 4G). Together, these Ca^2+^ imaging data hint at a Ca^2+^ influx defect in DISC1-inactivated cells, although abnormal clearance of Ca^2+^ from the presynaptic terminal could also be involved (see “Discussion” Section).

To determine whether reduced Ca^2+^ influx is the primary cause of SV cycling defects, we attempted to rescue SV exocytosis in DISC1-silenced neurons by elevating extracellular Ca^2+^ concentration. Shifting extracellular Ca^2+^ concentration from 2 to 4 mM increases AP-induced Ca^2+^ entry (Ariel and Ryan, 2010) and restored the vGpH response (Figure 4H), in line with a role of DISC1 in facilitating Ca^2+^ influx.

**Figure 5:**
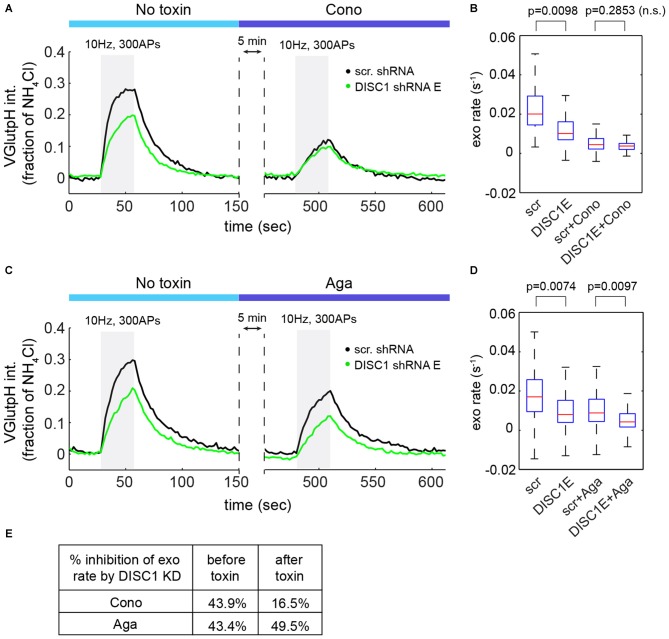
**DISC1 regulates Cav2.2-dependent SV exocytosis. (A)** Average vGpH traces in scr (101 boutons, 6 fields, 2 exps) and DISC1-E (87 boutons, 5 fields, 2 exps) shRNA-expressing neurons during consecutive trains of APs (300 AP, 10 Hz) in the absence or presence of the Cav2.2 blocker ω-Conotoxin GVIA (125 nM). **(B)** Boxplot of SV exocytic rates before and after ω-Conotoxin GVIA application. Exocytic rates were measured by linear fitting of the first six time points of the vGpH response. **(C)** Average vGpH traces in scr (1294 boutons, 9 fields, 3 exps) and DISC1-E (1282 boutons, 9 fields, 3 exps) shRNA-expressing neurons during consecutive trains of APs (300 AP, 10 Hz) in the absence or presence of the Cav2.1 blocker ω-Agatoxin TK (125 nM). **(D)** Boxplot of SV exocytic rates before and after ω-Agatoxin TK application. **(E)** Table showing the percentage of inhibition of exocytosis rate by DISC1 knockdown before and after Cav2.2- or Cav2.1 blockade.

At central synapses, the P/Q-type (Cav2.1) and N-type (Cav2.2) Ca^2+^ channels are the main sources of Ca^2+^ for initiation of SV exocytosis (Catterall et al., 2013). We used channel-specific toxins to determine the contribution of each subtype to SV release in hippocampal neurons. ω-Conotoxin GVIA (an N-type blocker) reduced SV exocytic rates by 73 ± 5% (Figures 5A,B), while ω-Agatoxin TK (a P/Q-type blocker) resulted in a 42 ± 3% decrease in SV exocytic rates (Figures 5C,D), in a good agreement with a recent study (Ariel et al., 2013). Note that in these experiments, exocytic rates were approximated by measuring the initial slope of the vGpH response in the absence of Baf (see Figure 1D). Importantly, blockade of Cav2.2 almost completely occluded the effect of DISC1 silencing on SV exocytosis (Figures 5A,B,E). Blockade of Cav2.1, on the other hand, slightly increased the inhibitory impact of DISC1 knockdown on SV release rates (Figures 5C–E). We conclude from these results that DISC1 selectively stimulates Cav2.2-dependent SV release at hippocampal synapses.

We then asked whether DISC1 regulates Cav2.2 activity by controlling its presynaptic localization and/or abundance. Co-localization studies of endogenous Cav2.2 with the active zone marker bassoon revealed extensive presence of Cav2.2 in synaptic boutons, both in control and DISC1-silenced neurons (Supplementary Figures S2A–C). We found no significant difference between these two groups in the fraction of boutons containing Cav2.2, or in the intensity of Cav2.2 staining in presynaptic terminals (Supplementary Figures S2B,C). Nor did we find evidence for altered presynaptic localization and intensity of Cav2.1 (Supplementary Figures S2D–F or reduced density of presynaptic terminals (Supplementary Figure S2G) in DISC1 knockdown neurons. Thus, synaptic targeting and abundance of Cav2 channels does not seem to be regulated by DISC1.

**Figure 6:**
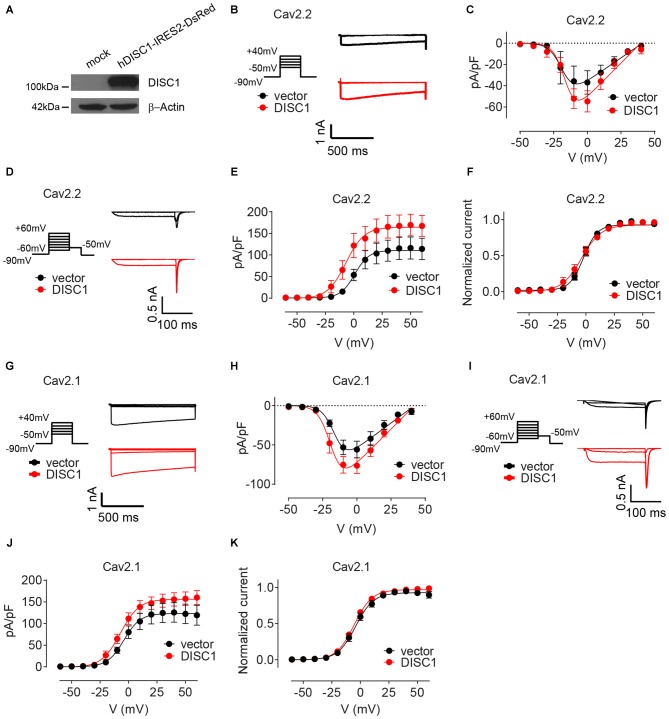
**DISC1 enhances Cav2.2 and Cav2.1 currents. (A)** Western blot showing expression of ectopic (human) DISC1 in HEK293 cells. **(B,C)** Cav2.2 Current-voltage (I-V) curves for hDISC1-expressing and control cells. **(B)** Stimulation protocol and individual current responses shown at three different voltages (−30, 0 and 30 mV) **(C)**. Average I-V plots for hDISC1 (peak = 54.2 ± 4.5 pA/pF, 13 cells) and control (peak = 39.2 ± 4.9 pA/pF, 12 cells), *p* = 0.033. **(D–F)** Cav2.2 activation curves in response to the tail protocol. **(D)** Illustration of the tail protocol and individual tail currents measured at −50 mV after three different voltage steps (−20, 0 and 40 mV). **(E)** Average current density based on tail currents for DISC1 (164.3 ± 7.3 pA/pF, 12 cells) and control (110.5 ± 6.3 pA/pF, 12 cells), *p < 0.001. **(F)** Normalized activation curve from tail currents showing no significant difference between DISC1 (V_50_: −6.98 ± 3.43 mV, 12 cells) and control (V_50_: 0.86 ± 3.62 mV, 12 cells), *p* = 0.13. **(G,H)** Cav2.1 Current-voltage (I-V) curves for hDISC1-expressing and control cells. **(G)** Stimulation protocol and individual current responses shown at three different voltages (−30, 0 and 30 mV). **(H)** Average I-V plots for hDISC1 (peak = 79.9 ± 8.3 pA/pF, 15 cells) and control (peak = 56.3 ± 7.4 pA/pF, 13 cells), *p* = 0.046. **(I–K)** Cav2.1 activation curves in response to the tail protocol. **(I)** Illustration of the tail protocol and individual tail currents measured at −50 mV after three different voltage steps (−20, 0 and 40 mV). **(J)** Average current density based on tail currents for DISC1 (156.3 ± 5.7 pA/pF, 25 cells) and control (123.2 ± 7.4 pA/pF, 17 cells), *p = 0.003. **(K)** Normalized activation curve from tail currents showing no significant difference between DISC1 (V_50_: −5.39 ± 0.49 mV, 24 cells) and control (V_50_: −4.41 ± 0.93 mV, 17 cells), *p* = 0.31.

We finally examined the effect of DISC1 on Cav2 currents by whole cell patch-clamp recordings. Co-transfection of recombinant DISC1 (Figure 6A) in HEK293T cells together with Cav2.2 and its auxiliary subunits (see “Materials and Methods” Section) resulted in a 27% increase in Cav2.2 peak current density (Figures 6B,C). Likewise, tail current density of Cav2.2 was substantially elevated in DISC1-overexpressing cells (Figures 6D,E). Current density can be affected by a change in channel gating or by the number of channels at the cell surface. To test the first possibility, we examined the voltage-dependent properties of Cav2.2 current activation and saw no difference between control and DISC1-overexpressing cells (Figure 6F). This suggests that DISC1 promotes surface delivery and/or stabilize surface expression of Cav2.2. Recording of Cav2.1 currents revealed a similar, voltage-independent, potentiation effect of DISC1 (Figures 6G–K). Thus, DISC1 equally augments both Cav2.2 and Cav2.1 currents in this heterologous system, presumably by increasing surface expression of these Ca^2+^ channels. These results also imply the presence of an additional layer of regulation in hippocampal neurons that restricts the activity of DISC1 to Cav2.2-dependent SV exocytosis.

## Discussion

We used here two independent gene-targeting approaches together with large-scale imaging of presynaptic function to determine the impact of the mental disease gene *DISC1* on the glutamate release machinery. Our results show that DISC1 accelerates SV exocytosis and thus enhances presynaptic performance. This boosting effect is mediated by N-type Ca^2+^ channels, establishing the first link between DISC1 and VGCC activity.

DISC1 inactivation in hippocampal neurons results in decreased Ca^2+^ transients at hippocampal terminals in response to AP firing, consistent with a deficit in Ca^2+^ entry. Because these Ca^2+^ signals are shaped by both Ca^2+^ entry and clearance processes, we cannot exclude a role of DISC1 in regulating Ca^2+^ extrusion or buffering. A stimulatory effect of DISC1 on Ca^2+^ entry is further supported, however, by its positive impact on Cav2.2 and Cav2.1 currents, the two main VGCCs underlying the initiation of neurotransmission at hippocampal synapses. How DISC1 enhances Cav2 currents is unclear. The lack of an effect on gating suggests that DISC1 promotes surface expression of these Ca^2+^ channels, although we cannot exclude a role of DISC1 in regulating single-channel conductance or open probability. Our immuno-localization studies revealed no detectable impact of DISC1 on presynaptic abundance of Cav2 channels. This does not rule out, however, the possibility that DISC1 regulates the number of functional Ca^2+^ channels at the surface of the terminal.

Although overexpression of DISC1 enhances both Cav2.1 and Cav2.2 currents in a heterologous system, our findings point to a selective influence of DISC1 on Cav2.2-dependent SV exocytosis. How is this selectivity achieved? It is possible that both Cav2.1 and Cav2.2 compete for DISC1 regulation at presynaptic terminals. The dominant contribution of Cav2.2 to SV release probably results (at least in part) from increased levels of Cav2.2 (relative to Cav2.1) at hippocampal terminals (Ariel et al., 2013). Thus, preferential regulation of Cav2.2 by endogenous levels of DISC1 combined with greater abundance of Cav2.2 may explain why DISC1-dependent SV exocytosis appears to be exclusively mediated by Cav2.2. Specificity could also arise, however, from functions of DISC1 downstream of Ca^2+^ entry. For example, DISC1 could be involved in positioning Cav2.2 in close proximity to the SV release machinery at the active zone, a process that dictates speed and fidelity of neurotransmission. While the role of Ca^2+^ entry in SV exocytosis is undisputed, its impact on SV endocytosis remains somewhat controversial. Recent evidence suggests, however, that Ca^2+^ couples rates of SV exo- and endocytosis and optimizes endocytic rates during AP bursts (Armbruster et al., 2013). We observed reduced endocytic rates in both DISC1-silenced and *DISC1*^Δ2–3/Δ2–3^ neurons. Although this effect was small and did not reach statistical significance, our results suggest that the influence of DISC1 on Cav2.2 impacts both exo- and endocytosis of SVs at hippocampal terminals.

Our findings extend on two recent studies implicating DISC1 in presynaptic function. In glutamatergic neurons differentiated from human iPS cells and derived from members of a family with a frameshift mutation in DISC1, deficits in SV release were observed after KCl-induced depolarization (Wen et al., 2014). In a separate report, RNAi knockdown of DISC1 in layer 2/3 neocortical neurons increases paired-pulse facilitation and appears to reduce probability of glutamate release (Maher and Loturco, 2012). Collectively, these results clearly identify DISC1 as a positive modulator of glutamate release.

The efficacy of neurotransmitter release determines not only the strength of synaptic excitation, but also dictates various forms of short-term plasticity (Abbott and Regehr, 2004), suggesting broad functions of DISC1 in synaptic computation and neural circuit performance. Although we have not directly measured release probability (P_r_), the impact of DISC1 on Ca^2+^ entry and SV cycling suggests a positive influence on P_r_ (see Maher and Loturco, 2012). Because synaptic facilitation and depression depend largely on the initial P_r_ (high P_r_ favors depression while low P_r_ favors facilitation), our results predict altered short-term plasticity in DISC1-deficient neural circuits. Of interest, abnormal short-term plasticity has been associated with deficits in moment-to-moment information processing and working memory, two hallmarks of schizophrenia (Crabtree and Gogos, 2014). Reduced efficacy in glutamate release may also be relevant for forms of long-term potentiation (LTP) that have clear presynaptic components, such as LTP at the perforant path-granule cell synapse in the dentate gyrus (Errington et al., 1987). Notably, this form of LTP is impaired in *DISC1*^Δ2–3/Δ2–3^ mice—a stronger tetanic stimulus is required for the expression of LTP (Kuroda et al., 2011). Our findings suggest that this LTP deficit could be due to a failure of the presynaptic terminal to undergo activity-dependent increase in release probability.

Several genes encoding VGCC subunits, including *CACNA1C, CACNB2* and *CACNA1I* have repeatedly been associated with schizophrenia and other psychiatric disorders (Ferreira et al., 2008; Cross-Disorder Group of the Psychiatric Genomics, 2013; Hamshere et al., 2013; Ripke et al., 2013; Schizophrenia Working Group of the Psychiatric Genomics, 2014). Although *CACNA1A* (Cav2.1) and *CACNA1B* (Cav2.2) are not typically associated with risk loci, *RIM1* (also called *RIMS1*)—a presynaptic scaffold that regulates density of P/Q- and N-type Ca^2+^ channels and SV docking at the active zone (Han et al., 2011)—was recently identified as a candidate gene for schizophrenia in the largest GWAS study conducted to date (Schizophrenia Working Group of the Psychiatric Genomics, 2014). Together these findings point to neurotransmitter release as a central process targeted in schizophrenia.

In conclusion, our results shed light on a novel mechanism by which a major susceptibility gene for mental illness enhances the efficacy of glutamate release, and provide further support for a central role of glutamate neurotransmission in schizophrenia and other major mood disorders.

## Author Contributions

WT and JVT performed and analyzed all imaging and biochemical experiments. QL performed and analyzed whole-cell patch-clamp recordings. MB carried out the statistical analysis. KBL made the original observation implicating DISC1 in SV cycling. KKu and KKa made the *DISC1* Δ2–3 mouse and the DISC1 C-terminus antibody. WT and MF wrote Matlab scripts. TWS and MF supervised the project. MF wrote the article.

## Conflict of Interest Statement

The authors declare that the research was conducted in the absence of any commercial or financial relationships that could be construed as a potential conflict of interest.

## Supplementary Material

FIGURE S1 **Variability of SV exocytic responses.** Scatter plot showing SV exocytic rates in individual boutons across six (color-coded) independent preparations. Each preparation comprises three fields colored light to dark. These rates were measured in hippocampal neurons (DIV14–16) expressing scr shRNA and correspond to the data shown in Figure 2.

FIGURE S2 **Presynaptic localization and abundance of Cav2 channels are unaffected by DISC1 silencing. (A,D)** Immunolocalization of endogenous Cav2.2 **(A)** or Cav2.1 **(D)** and bassoon in rat hippocampal neurons (DIV 14–15) expressing scr and DISC1-E shRNAs. Arrowheads indicate boutons positive for Cav2.2 and bassoon **(A)** or Cav2.1 and bassoon **(D)**. Scale bar 10 μm. **(B)** Percentage of Cav2.2-positive boutons in scr (5 cells) and DISC1-E (5 cells) shRNA-expressing neurons. **(C)** Cav2.2 fluorescence intensity measured at individual presynaptic boutons (based on bassoon staining) in **(B)**. **(E)** Percentage of Cav2.1-positive boutons in scr (8 cells) and DISC1-E (8 cells) shRNA-expressing neurons. **(F)** Cav2.1 fluorescence intensity measured at individual presynaptic boutons (based on bassoon staining) in **(E)**. **(G)** Quantification of the density of presynaptic terminals (based on bassoon staining) in neurons expressing scr (24 cells), DISC1–E (21 cells) and -A (17 cells) shRNAs from four independent experiments.

TABLE T1 **Statistical analysis of synaptic responses.** Column 1: datasets. Column 2: *p* values derived from the field averaging approach, with each field weighted according to its number of boutons (see “Materials and Methods” Section). Column 3: *p* values derived from the linear mixed model, where intra-field correlations and variations in neuron preparations are taken into account (see “Materials and Methods” Section).

## References

[B1] Abbott L. F. Regehr W. G. (2004). Synaptic computation. Nature 431, 796–803. 10.1038/nature03010 15483601

[B2] Akerboom J. Chen T. W. Wardill T. J. Tian L. Marvin J. S. Mutlu S.. (2012). Optimization of a GCaMP calcium indicator for neural activity imaging. J. Neurosci. 32, 13819–13840. 10.1523/jneurosci.2601-12.2012 23035093 PMC3482105

[B4] Ariel P. Hoppa M. B. Ryan T. A. (2013). Intrinsic variability in Pv, RRP size, Ca^2+^ channel repertoire and presynaptic potentiation in individual synaptic boutons. Front. Synaptic Neurosci. 4:9. 10.3389/fnsyn.2012.00009 23335896 PMC3542534

[B3] Ariel P. Ryan T. A. (2010). Optical mapping of release properties in synapses. Front. Neural Circuits 4:18. 10.3389/fncir.2010.00018 20802854 PMC2928663

[B5] Armbruster M. Messa M. Ferguson S. M. De Camilli P. Ryan T. A. (2013). Dynamin phosphorylation controls optimization of endocytosis for brief action potential bursts. Elife 2:e00845. 10.7554/elife.00845 23908769 PMC3728620

[B6] Burrone J. Li Z. Murthy V. N. (2006). Studying vesicle cycling in presynaptic terminals using the genetically encoded probe synaptopHluorin. Nat. Protoc. 1, 2970–2978. 10.1038/nprot.2006.449 17406557

[B7] Catterall W. A. Leal K. Nanou E. (2013). Calcium channels and short-term synaptic plasticity. J. Biol. Chem. 288, 10742–10749. 10.1074/jbc.r112.411645 23400776 PMC3624454

[B8] Chubb J. E. Bradshaw N. J. Soares D. C. Porteous D. J. Millar J. K. (2008). The DISC locus in psychiatric illness. Mol. Psychiatry 13, 36–64. 10.1038/sj.mp.4002106 17912248

[B9] Clapcote S. J. Lipina T. V. Millar J. K. Mackie S. Christie S. Ogawa F.. (2007). Behavioral phenotypes of Disc1 missense mutations in mice. Neuron 54, 387–402. 10.1016/j.neuron.2007.04.015 17481393

[B10] Crabtree G. W. Gogos J. A. (2014). Synaptic plasticity, neural circuits and the emerging role of altered short-term information processing in schizophrenia. Front. Synaptic Neurosci. 6:28. 10.3389/fnsyn.2014.00028 25505409 PMC4243504

[B11] Cross-Disorder Group of the Psychiatric Genomics C. (2013). Identification of risk loci with shared effects on five major psychiatric disorders: a genome-wide analysis. Lancet 381, 1371–1379. 10.1016/s0140-6736(12)62129-1 23453885 PMC3714010

[B12] Cross-Disorder Group of the Psychiatric Genomics C. Lee S. H. Ripke S. Neale B. M. Faraone S. V. Purcell S. M.. (2013). Genetic relationship between five psychiatric disorders estimated from genome-wide SNPs. Nat. Genet. 45, 984–994. 10.1038/ng.2711 23933821 PMC3800159

[B13] Duan X. Chang J. H. Ge S. Faulkner R. L. Kim J. Y. Kitabatake Y.. (2007). Disrupted-In-Schizophrenia 1 regulates integration of newly generated neurons in the adult brain. Cell 130, 1146–1158. 10.1016/j.cell.2007.07.010 17825401 PMC2002573

[B14] Ekelund J. Hennah W. Hiekkalinna T. Parker A. Meyer J. Lonnqvist J.. (2004). Replication of 1q42 linkage in Finnish schizophrenia pedigrees. Mol. Psychiatry 9, 1037–1041. 10.1038/sj.mp.4001536 15197400

[B15] Ekelund J. Hovatta I. Parker A. Paunio T. Varilo T. Martin R.. (2001). Chromosome 1 loci in Finnish schizophrenia families. Hum. Mol. Genet. 10, 1611–1617. 10.1093/hmg/10.15.1611 11468279

[B16] Errington M. L. Lynch M. A. Bliss T. V. (1987). Long-term potentiation in the dentate gyrus: induction and increased glutamate release are blocked by D(-)aminophosphonovalerate. Neuroscience 20, 279–284. 10.1016/0306-4522(87)90019-4 2882444

[B17] Fernandez-Alfonso T. Ryan T. A. (2004). The kinetics of synaptic vesicle pool depletion at CNS synaptic terminals. Neuron 41, 943–953. 10.1016/s0896-6273(04)00113-8 15046726

[B18] Ferreira M. A. O’donovan M. C. Meng Y. A. Jones I. R. Ruderfer D. M. Jones L.. (2008). Collaborative genome-wide association analysis supports a role for ANK3 and CACNA1C in bipolar disorder. Nat. Genet. 40, 1056–1058. 10.1038/ng.209 18711365 PMC2703780

[B19] Fromer M. Pocklington A. J. Kavanagh D. H. Williams H. J. Dwyer S. Gormley P.. (2014). De novo mutations in schizophrenia implicate synaptic networks. Nature 506, 179–184. 10.1038/nature12929 24463507 PMC4237002

[B20] Galbraith S. Daniel J. A. Vissel B. (2010). A study of clustered data and approaches to its analysis. J. Neurosci. 30, 10601–10608. 10.1523/jneurosci.0362-10.2010 20702692 PMC6634702

[B21] Garcia-Alvarez G. Lu B. Yap K. A. Wong L. C. Thevathasan J. V. Lim L.. (2015). STIM2 regulates PKA-dependent phosphorylation and trafficking of AMPARs. Mol. Biol. Cell 26, 1141–1159. 10.1091/mbc.e14-07-1222 25609091 PMC4357513

[B22] Glessner J. T. Reilly M. P. Kim C. E. Takahashi N. Albano A. Hou C.. (2010). Strong synaptic transmission impact by copy number variations in schizophrenia. Proc. Natl. Acad. Sci. U S A 107, 10584–10589. 10.1073/pnas.1000274107 20489179 PMC2890845

[B23] Hamshere M. L. Walters J. T. Smith R. Richards A. L. Green E. Grozeva D.. (2013). Genome-wide significant associations in schizophrenia to ITIH3/4, CACNA1C and SDCCAG8 and extensive replication of associations reported by the Schizophrenia PGC. Mol. Psychiatry 18, 708–712. 10.1038/mp.2012.67 22614287 PMC4724864

[B24] Han Y. Kaeser P. S. Sudhof T. C. Schneggenburger R. (2011). RIM determines Ca^2+^ channel density and vesicle docking at the presynaptic active zone. Neuron 69, 304–316. 10.1016/j.neuron.2010.12.014 21262468 PMC3259453

[B25] Hikida T. Jaaro-Peled H. Seshadri S. Oishi K. Hookway C. Kong S.. (2007). Dominant-negative DISC1 transgenic mice display schizophrenia-associated phenotypes detected by measures translatable to humans. Proc. Natl. Acad. Sci. U S A 104, 14501–14506. 10.1073/pnas.0704774104 17675407 PMC1964873

[B26] Howes O. Mccutcheon R. Stone J. (2015). Glutamate and dopamine in schizophrenia: an update for the 21st century. J. Psychopharmacol. 29, 97–115. 10.1177/0269881114563634 25586400 PMC4902122

[B27] Huang H. Tan B. Z. Shen Y. Tao J. Jiang F. Sung Y. Y.. (2012). RNA editing of the IQ domain in Ca(v)1.3 channels modulates their Ca^2+^-dependent inactivation. Neuron 73, 304–316. 10.1016/j.neuron.2011.11.022 22284185 PMC3271027

[B28] Huettner J. E. Baughman R. W. (1986). Primary culture of identified neurons from the visual cortex of postnatal rats. J. Neurosci. 6, 3044–3060. 3760948 10.1523/JNEUROSCI.06-10-03044.1986 PMC6568785

[B29] International Schizophrenia C. Purcell S. M. Wray N. R. Stone J. L. Visscher P. M. O’donovan M. C.. (2009). Common polygenic variation contributes to risk of schizophrenia and bipolar disorder. Nature 460, 748–752. 10.1038/nature08185 19571811 PMC3912837

[B30] Kaech S. Banker G. (2006). Culturing hippocampal neurons. Nat. Protoc. 1, 2406–2415. 10.1038/nprot.2006.356 17406484

[B31] Kamiya A. Kubo K. Tomoda T. Takaki M. Youn R. Ozeki Y.. (2005). A schizophrenia-associated mutation of DISC1 perturbs cerebral cortex development. Nat. Cell Biol. 7, 1167–1178. 10.1038/ncb1328 16299498

[B32] Kilpinen H. Ylisaukko-Oja T. Hennah W. Palo O. M. Varilo T. Vanhala R.. (2008). Association of DISC1 with autism and Asperger syndrome. Mol. Psychiatry 13, 187–196. 10.1038/sj.mp.4002031 17579608

[B33] Kubo K. Tomita K. Uto A. Kuroda K. Seshadri S. Cohen J.. (2010). Migration defects by DISC1 knockdown in C57BL/6, 129X1/SvJ and ICR strains via in utero gene transfer and virus-mediated RNAi. Biochem. Biophys. Res. Commun. 400, 631–637. 10.1016/j.bbrc.2010.08.117 20807500 PMC2949544

[B34] Kuroda K. Yamada S. Tanaka M. Iizuka M. Yano H. Mori D.. (2011). Behavioral alterations associated with targeted disruption of exons 2 and 3 of the Disc1 gene in the mouse. Hum. Mol. Genet. 20, 4666–4683. 10.1093/hmg/ddr400 21903668

[B35] Laird N. M. Ware J. H. (1982). Random-effects models for longitudinal data. Biometrics 38, 963–974. 7168798

[B36] Lee F. H. Fadel M. P. Preston-Maher K. Cordes S. P. Clapcote S. J. Price D. J.. (2011). Disc1 point mutations in mice affect development of the cerebral cortex. J. Neurosci. 31, 3197–3206. 10.1523/jneurosci.4219-10.2011 21368031 PMC6623921

[B37] Li H. Foss S. M. Dobryy Y. L. Park C. K. Hires S. A. Shaner N. C.. (2011). Concurrent imaging of synaptic vesicle recycling and calcium dynamics. Front. Mol. Neurosci. 4:34. 10.3389/fnmol.2011.00034 22065946 PMC3206542

[B38] Maher B. J. Loturco J. J. (2012). Disrupted-in-schizophrenia (DISC1) functions presynaptically at glutamatergic synapses. PLoS One 7:e34053. 10.1371/journal.pone.0034053 22479520 PMC3316587

[B39] Mao Y. Ge X. Frank C. L. Madison J. M. Koehler A. N. Doud M. K.. (2009). Disrupted in schizophrenia 1 regulates neuronal progenitor proliferation via modulation of GSK3beta/beta-catenin signaling. Cell 136, 1017–1031. 10.1016/j.cell.2008.12.044 19303846 PMC2704382

[B40] Miesenbock G. De Angelis D. A. Rothman J. E. (1998). Visualizing secretion and synaptic transmission with pH-sensitive green fluorescent proteins. Nature 394, 192–195. 10.1038/28190 9671304

[B41] Millar J. K. Christie S. Anderson S. Lawson D. Hsiao-Wei Loh D. Devon R. S.. (2001). Genomic structure and localisation within a linkage hotspot of Disrupted In Schizophrenia 1, a gene disrupted by a translocation segregating with schizophrenia. Mol. Psychiatry 6, 173–178. 10.1038/sj.mp.4000784 11317219

[B42] Millar J. K. Pickard B. S. Mackie S. James R. Christie S. Buchanan S. R.. (2005). DISC1 and PDE4B are interacting genetic factors in schizophrenia that regulate cAMP signaling. Science 310, 1187–1191. 10.1126/science.1112915 16293762

[B43] Millar J. K. Wilson-Annan J. C. Anderson S. Christie S. Taylor M. S. Semple C. A.. (2000). Disruption of two novel genes by a translocation co-segregating with schizophrenia. Hum. Mol. Genet. 9, 1415–1423. 10.1093/hmg/9.9.1415 10814723

[B44] Moskvina V. Craddock N. Holmans P. Nikolov I. Pahwa J. S. Green E.. (2009). Gene-wide analyses of genome-wide association data sets: evidence for multiple common risk alleles for schizophrenia and bipolar disorder and for overlap in genetic risk. Mol. Psychiatry 14, 252–260. 10.1038/mp.2008.133 19065143 PMC3970088

[B45] Niwa M. Kamiya A. Murai R. Kubo K. Gruber A. J. Tomita K.. (2010). Knockdown of DISC1 by in utero gene transfer disturbs postnatal dopaminergic maturation in the frontal cortex and leads to adult behavioral deficits. Neuron 65, 480–489. 10.1016/j.neuron.2010.01.019 20188653 PMC3084528

[B46] Pletnikov M. V. Ayhan Y. Nikolskaia O. Xu Y. Ovanesov M. V. Huang H.. (2008). Inducible expression of mutant human DISC1 in mice is associated with brain and behavioral abnormalities reminiscent of schizophrenia. Mol. Psychiatry 13, 173–186. 10.1038/sj.mp.4002079 17848917

[B47] Poon V. Y. Goh C. Voorhoeve P. M. Fivaz M. (2014). High-content imaging of presynaptic assembly. Front. Cell Neurosci. 8:66. 10.3389/fncel.2014.00066 24624059 PMC3939450

[B48] Ripke S. O’dushlaine C. Chambert K. Moran J. L. Kahler A. K. Akterin S.. (2013). Genome-wide association analysis identifies 13 new risk loci for schizophrenia. Nat. Genet. 45, 1150–1159. 10.1038/ng.2742 23974872 PMC3827979

[B49] Sachs N. A. Sawa A. Holmes S. E. Ross C. A. Delisi L. E. Margolis R. L. (2005). A frameshift mutation in Disrupted in Schizophrenia 1 in an American family with schizophrenia and schizoaffective disorder. Mol. Psychiatry 10, 758–764. 10.1038/sj.mp.4001667 15940305

[B50] Sankaranarayanan S. De Angelis D. Rothman J. E. Ryan T. A. (2000). The use of pHluorins for optical measurements of presynaptic activity. Biophys. J. 79, 2199–2208. 10.1016/s0006-3495(00)76468-x 11023924 PMC1301110

[B51] Schizophrenia Working Group of the Psychiatric Genomics C. (2014). Biological insights from 108 schizophrenia-associated genetic loci. Nature 511, 421–427. 10.1038/nature13595 25056061 PMC4112379

[B52] Shen S. Lang B. Nakamoto C. Zhang F. Pu J. Kuan S. L.. (2008). Schizophrenia-related neural and behavioral phenotypes in transgenic mice expressing truncated Disc1. J. Neurosci. 28, 10893–10904. 10.1523/jneurosci.3299-08.2008 18945897 PMC6671369

[B53] Shinoda T. Taya S. Tsuboi D. Hikita T. Matsuzawa R. Kuroda S.. (2007). DISC1 regulates neurotrophin-induced axon elongation via interaction with Grb2. J. Neurosci. 27, 4–14. 10.1523/jneurosci.3825-06.2007 17202467 PMC6672285

[B54] Singh K. K. Ge X. Mao Y. Drane L. Meletis K. Samuels B. A.. (2010). Dixdc1 is a critical regulator of DISC1 and embryonic cortical development. Neuron 67, 33–48. 10.1016/j.neuron.2010.06.002 20624590 PMC2938013

[B55] St Clair D. Blackwood D. Muir W. Carothers A. Walker M. Spowart G.. (1990). Association within a family of a balanced autosomal translocation with major mental illness. Lancet 336, 13–16. 10.1016/0140-6736(90)91520-k 1973210

[B56] Steinecke A. Gampe C. Valkova C. Kaether C. Bolz J. (2012). Disrupted-in-Schizophrenia 1 (DISC1) is necessary for the correct migration of cortical interneurons. J. Neurosci. 32, 738–745. 10.1523/jneurosci.5036-11.2012 22238109 PMC6621064

[B57] Thevathasan J. V. Tan E. Zheng H. Lin Y. C. Li Y. Inoue T.. (2013). The small GTPase HRas shapes local PI3K signals through positive feedback and regulates persistent membrane extension in migrating fibroblasts. Mol. Biol. Cell 24, 2228–2237. 10.1091/mbc.E12-12-0905 23676667 PMC3708728

[B58] Tian L. Hires S. A. Mao T. Huber D. Chiappe M. E. Chalasani S. H.. (2009). Imaging neural activity in worms, flies and mice with improved GCaMP calcium indicators. Nat. Methods 6, 875–881. 10.1038/nmeth.1398 19898485 PMC2858873

[B59] Tiscornia G. Singer O. Verma I. M. (2006). Production and purification of lentiviral vectors. Nat. Protoc. 1, 241–245. 10.1038/nprot.2006.37 17406239

[B60] Tsuboi D. Kuroda K. Tanaka M. Namba T. Iizuka Y. Taya S.. (2015). Disrupted-in-schizophrenia 1 regulates transport of ITPR1 mRNA for synaptic plasticity. Nat. Neurosci. 18, 698–707. 10.1038/nn.3984 25821909

[B61] Voglmaier S. M. Kam K. Yang H. Fortin D. L. Hua Z. Nicoll R. A.. (2006). Distinct endocytic pathways control the rate and extent of synaptic vesicle protein recycling. Neuron 51, 71–84. 10.1016/j.neuron.2006.05.027 16815333

[B62] Wen Z. Nguyen H. N. Guo Z. Lalli M. A. Wang X. Su Y.. (2014). Synaptic dysregulation in a human iPS cell model of mental disorders. Nature 515, 414–418. 10.1038/nature13716 25132547 PMC4501856

