# Supplementary figures and images for: Stimulation of Synaptic Vesicle Exocytosis by the Mental Disease Gene DISC1 is Mediated by N-Type Voltage-Gated Calcium Channels
Source: Front Synaptic Neurosci. 2016 Jun 14;8:15. doi: 10.3389/fnsyn.2016.00015 (PMC4906242; doi:10.3389/fnsyn.2016.00015)

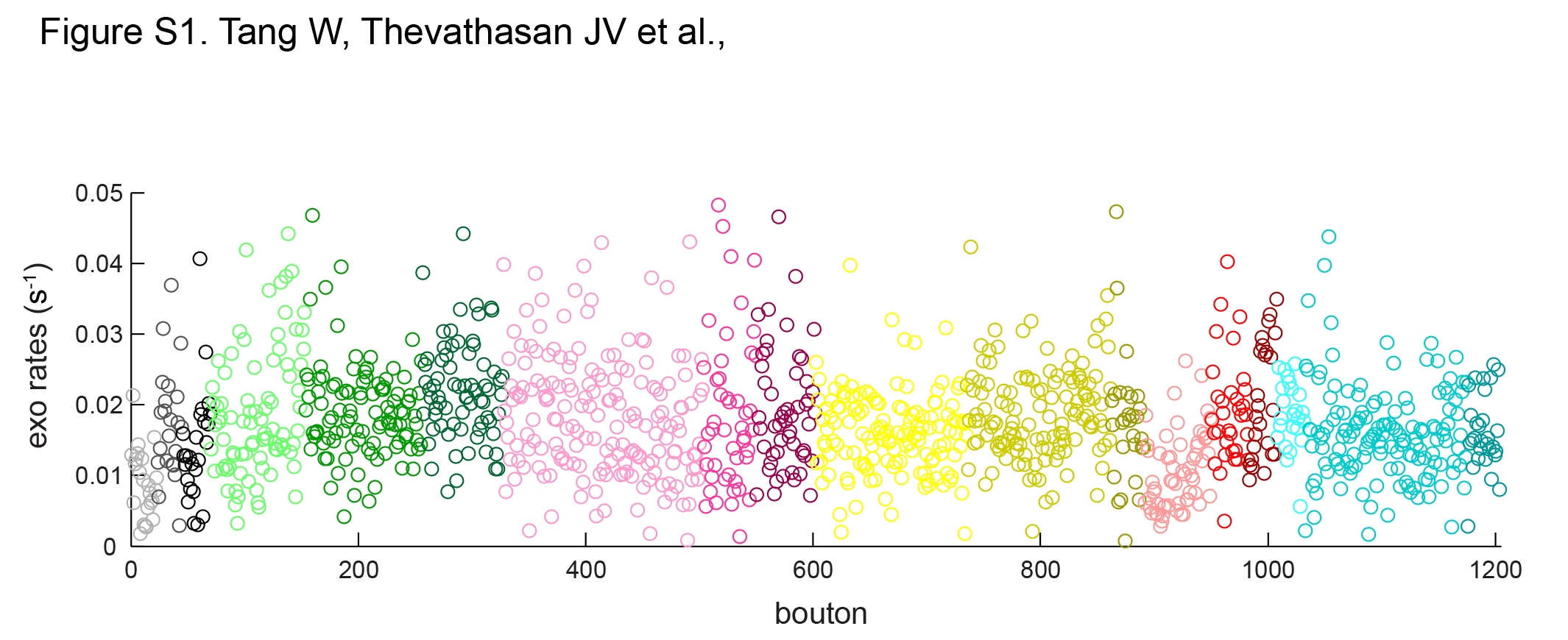

Supplement: FIGURE S1 — Variability of SV exocytic responses. Scatter plot showing SV exocytic rates in individual boutons across six (color-coded) independent preparations. Each preparation comprises three fields colored light to dark. These rates were measured in hippocampal neurons (DIV14–16) expressing scr shRNA and correspond to the data shown in Figure 2. [file Image_1.tif]

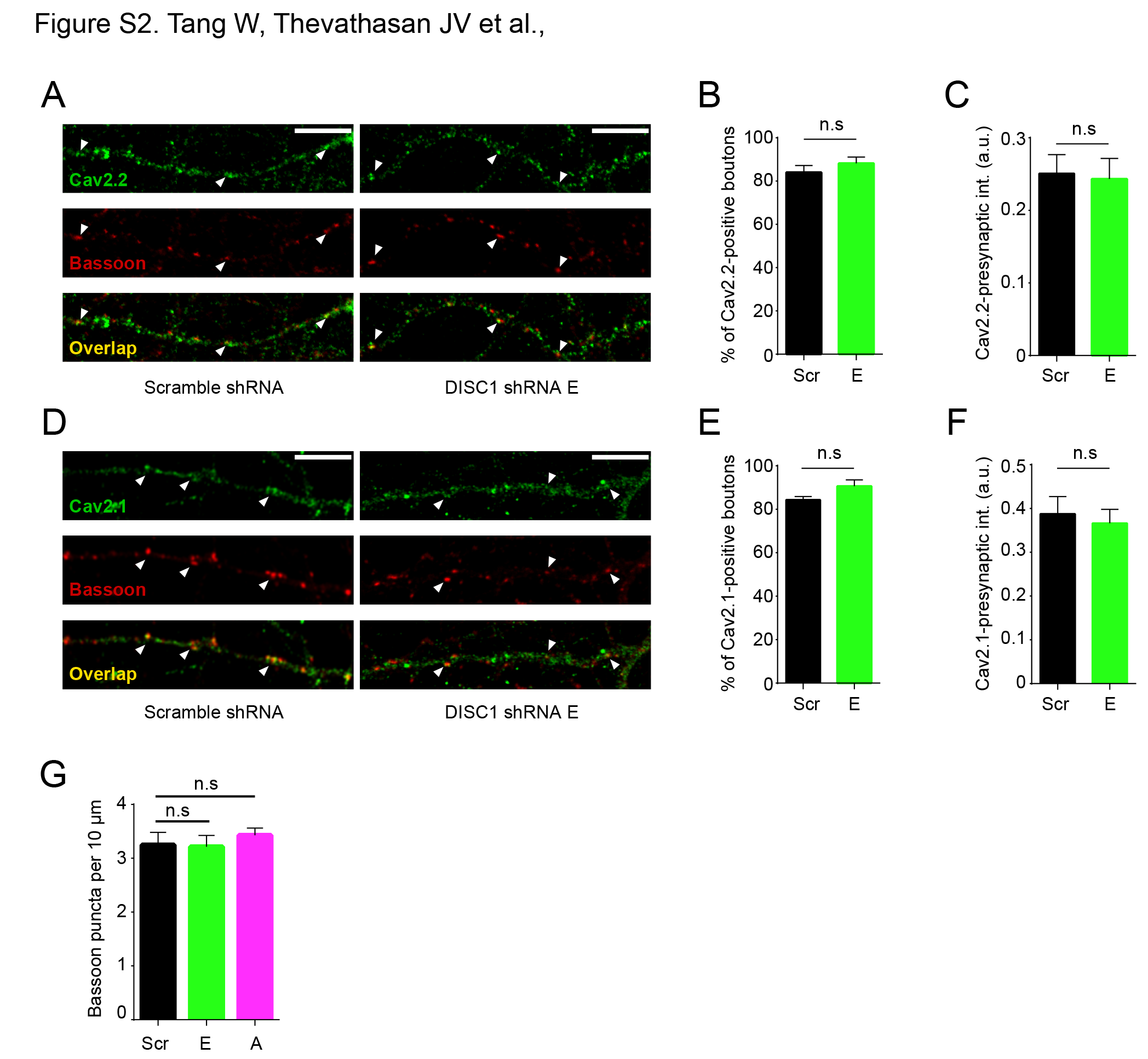

Supplement: FIGURE S2 — Presynaptic localization and abundance of Cav2 channels are unaffected by DISC1 silencing. (A,D) Immunolocalization of endogenous Cav2.2 (A) or Cav2.1 (D) and bassoon in rat hippocampal neurons (DIV 14–15) expressing scr and DISC1-E shRNAs. Arrowheads indicate boutons positive for Cav2.2 and bassoon (A) or Cav2.1 and bassoon (D). Scale bar 10 μm. (B) Percentage of Cav2.2-positive boutons in scr (5 cells) and DISC1-E (5 cells) shRNA-expressing neurons. (C) Cav2.2 fluorescence intensity measured at individual presynaptic boutons (based on bassoon staining) in (B). (E) Percentage of Cav2.1-positive boutons in scr (8 cells) and DISC1-E (8 cells) shRNA-expressing neurons. (F) Cav2.1 fluorescence intensity measured at individual presynaptic boutons (based on bassoon staining) in (E). (G) Quantification of the density of presynaptic terminals (based on bassoon staining) in neurons expressing scr (24 cells), DISC1–E (21 cells) and -A (17 cells) shRNAs from four independent experiments. [file Image_2.tif]
